# Supplementary material for: A cooperative nano-CRISPR scaffold potentiates immunotherapy via activation of tumour-intrinsic pyroptosis
Source: Nat Commun. 2023 Feb 11;14:779. doi: 10.1038/s41467-023-36550-9 (PMC9922300; doi:10.1038/s41467-023-36550-9)
Supplement: Supplementary file 1 — Supplementary Information [file 41467_2023_36550_MOESM1_ESM.pdf]

Supplementary Information for

A Cooperative Nano-CRISPR Scaffold Potentiates Immunotherapy via Activation of Tumour-intrinsic Pyroptosis

Ning Wang [1†], Chao Liu [1†], Yingjie Li [1], Dongxue Huang [1], Xinyue Wu [1], Xiaorong Kou [1], Xiye Wang [1], Qinjie Wu [1] *, Changyang Gong [1] *

[1] State Key Laboratory of Biotherapy and Cancer Center, West China Hospital, Sichuan University, Chengdu, 610041, P. R. China

[†] These authors contributed equally to this work

[*] To whom correspondence should be addressed (C Gong, Q Wu). E-mail: chygong14@163.com, cellwqj@163.com.

Supplementary Figures

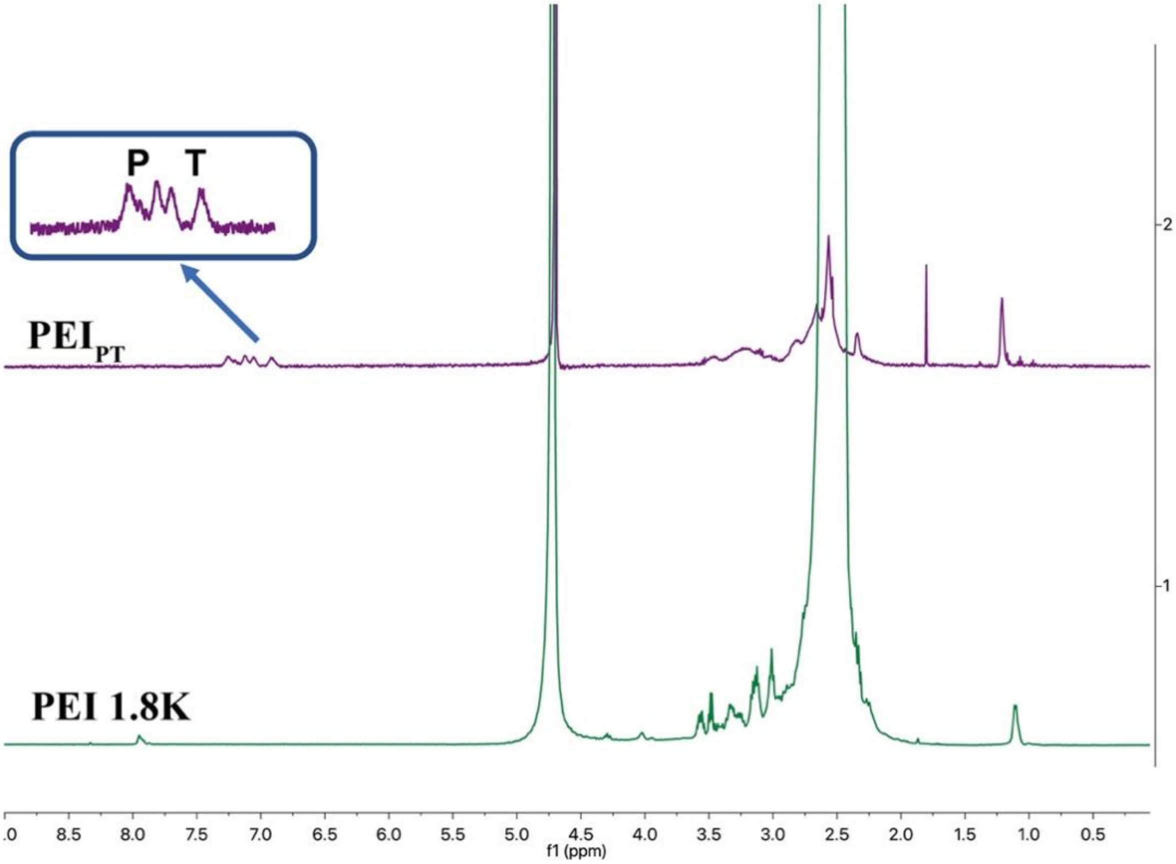

Supplementary Fig. 1. ^{1}H NMR spectrum of PEI_{PT} in D_2O.

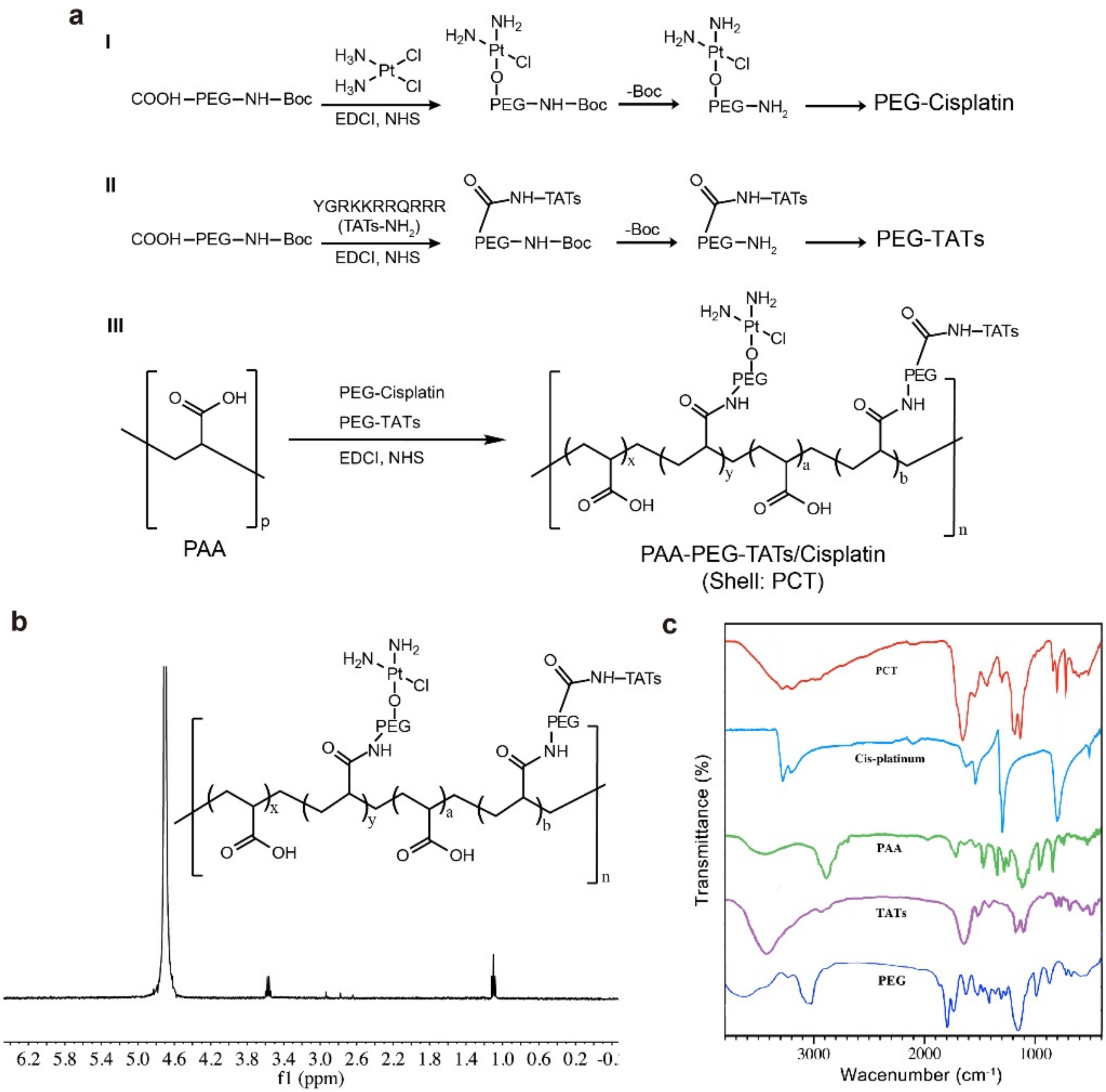

Supplementary Fig. 2. Synthesis and characterization of PCT. a, Synthesis routines of PCT. **b**, Spectrum of PCT in D_2O. **c**, FT-IR of PCT.

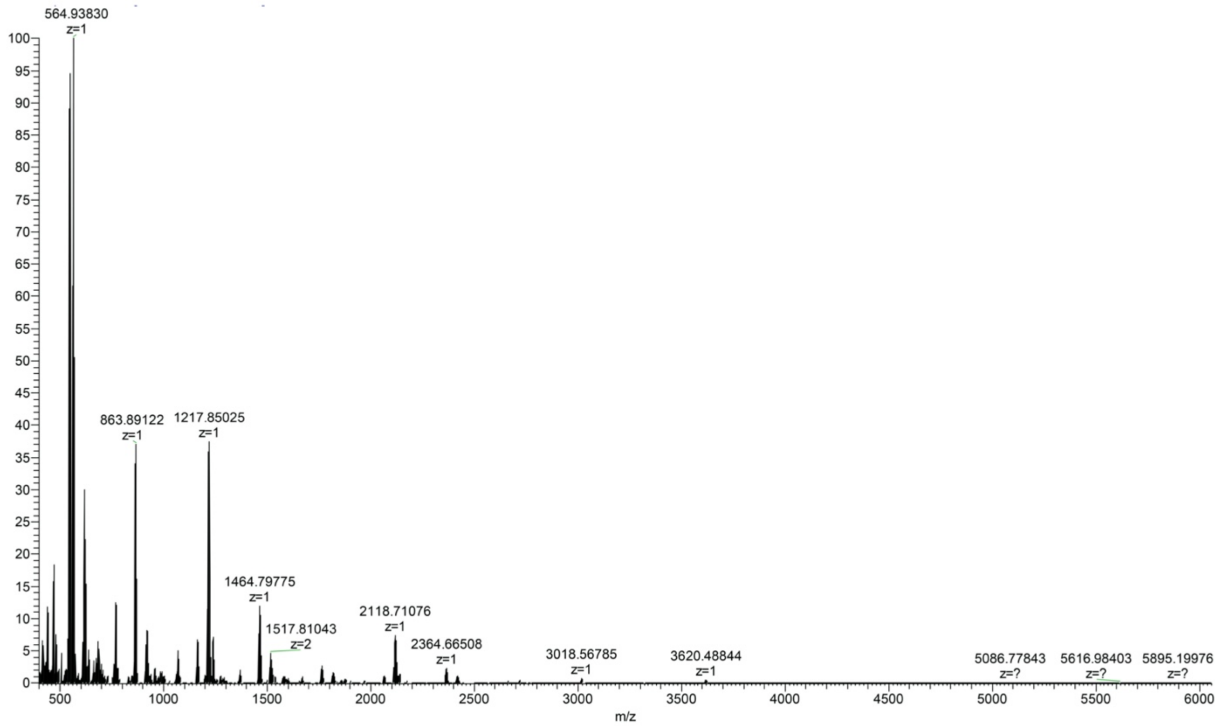

Supplementary Fig. 3. Mass spectrum of PCT.

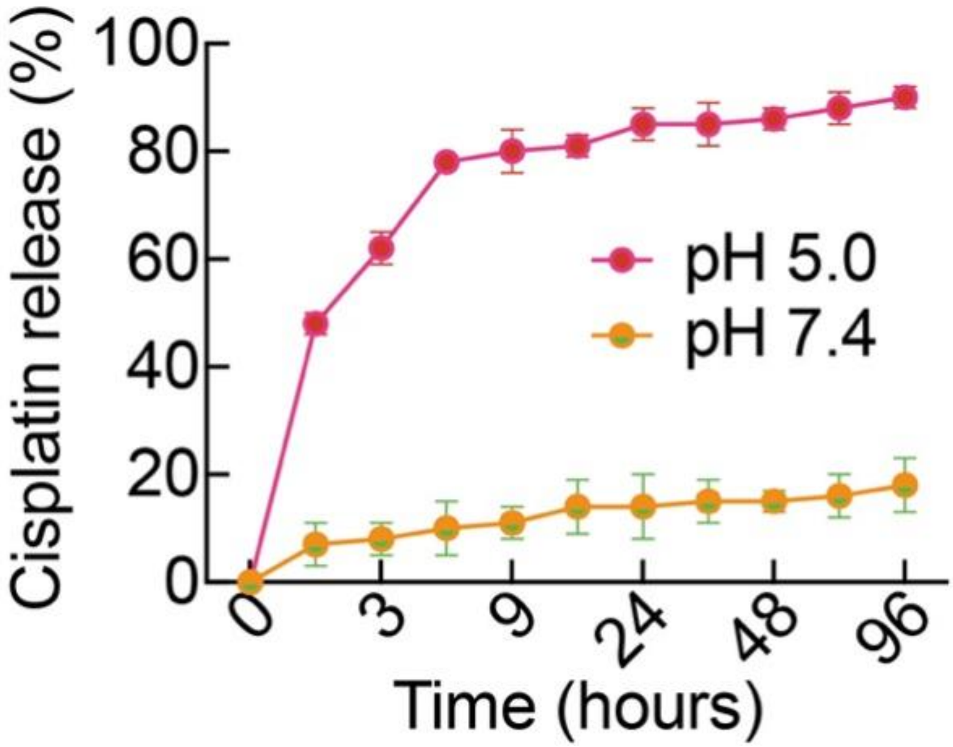

Supplementary Fig. 4. Cisplatin release behavior under pH 5.0 or pH 7.4 at different time points by HPLC. Data are presented as the mean ± s.d. (n = 3 biological replicates per group).

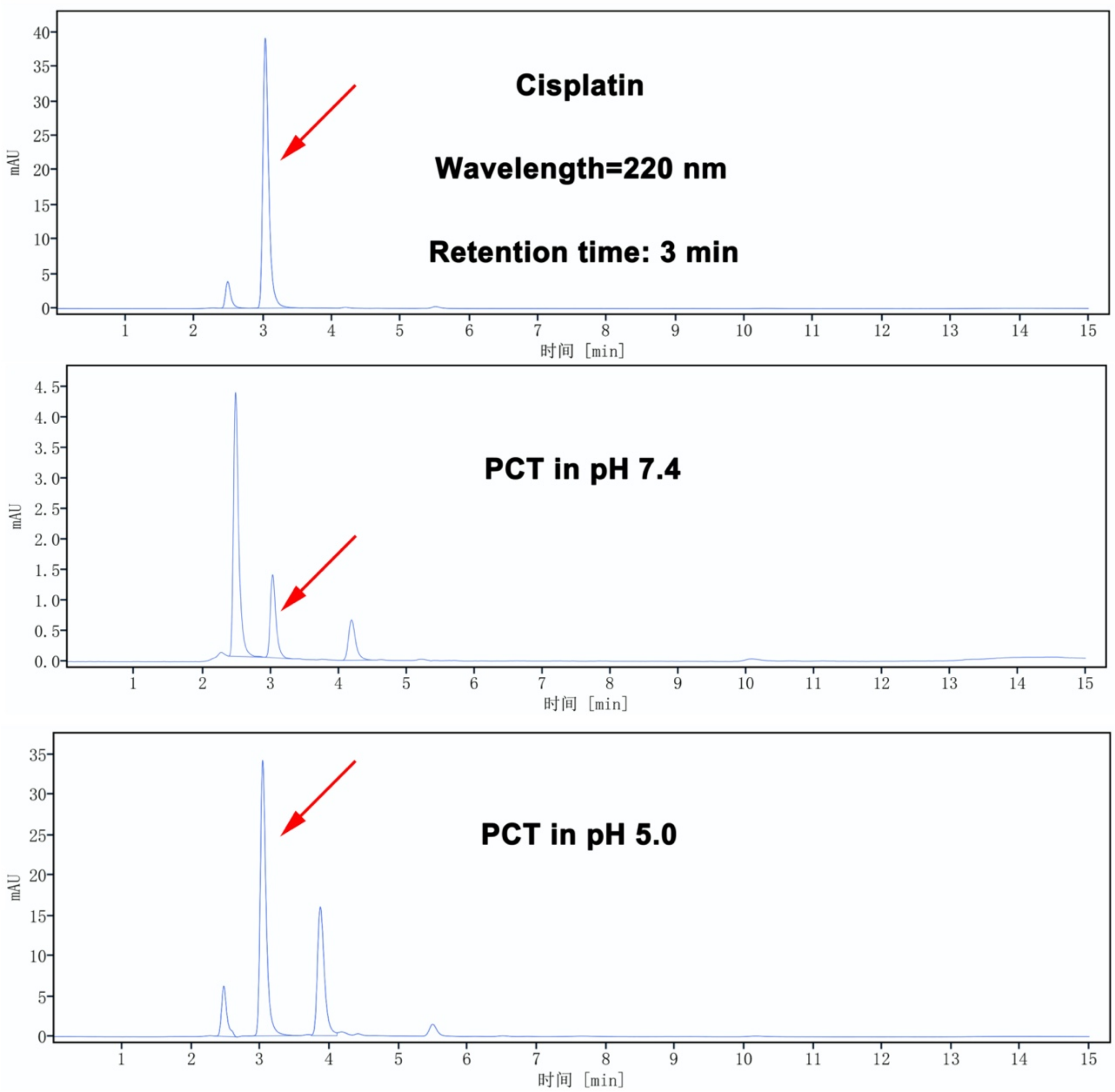

Supplementary Fig. 5. HPLC spectrum of Cisplatin, PCT in pH 7.4 for 24 h or PCT in pH 5.0 for 24 h.

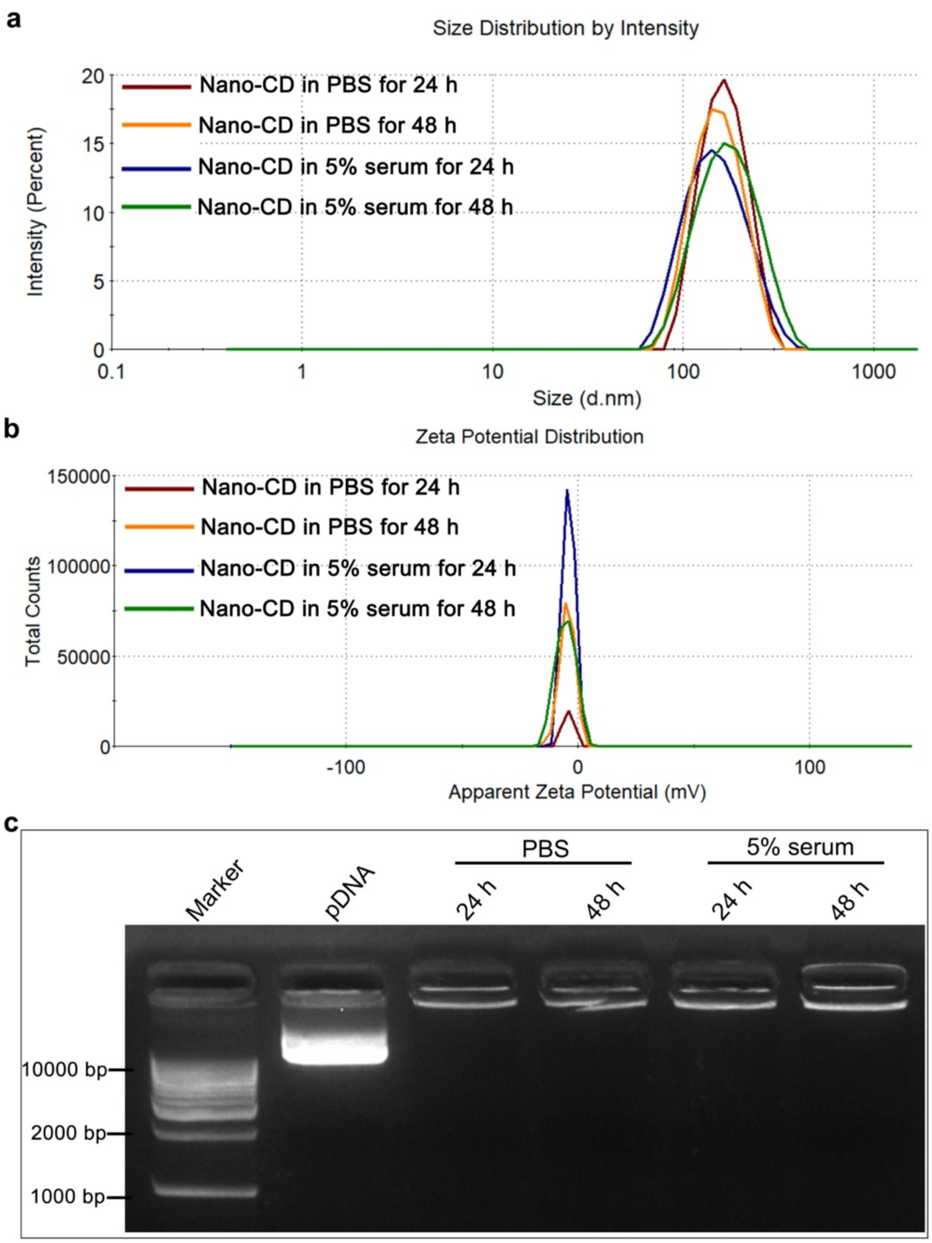

Supplementary Fig. 6. The stability of Nano-CD in PBS or 5% serum for 24h or 48 h. **a,b**, The particle size and zeta potential of Nano-CD in PBS or 5% serum for 24 h or 48 h. **c**, Agarose gel electrophoresis of Nano-CD in PBS or 5% serum for 24 h or 48 h (n =3).

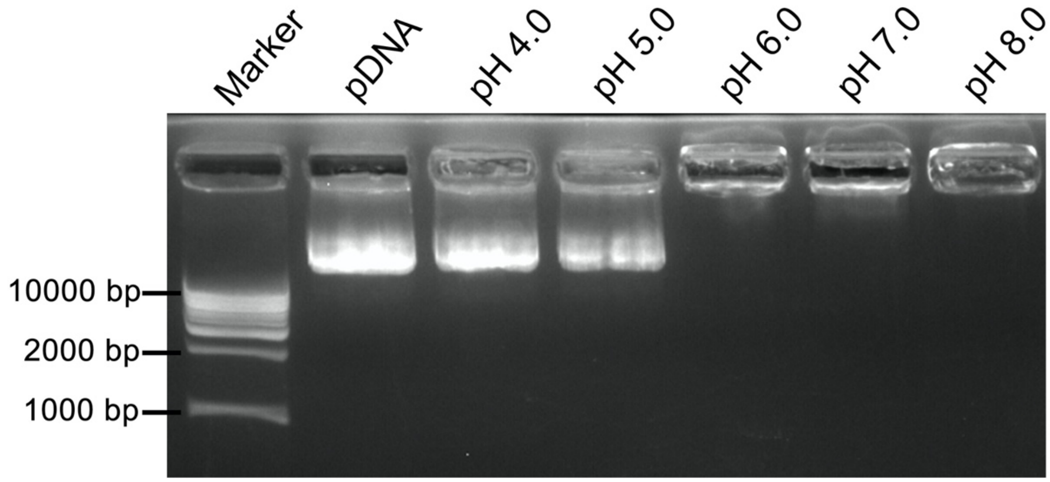

Supplementary Fig. 7. Agarose gel electrophoresis of Nano-CD in different pH (pH 4.0-8.0) (n =3).

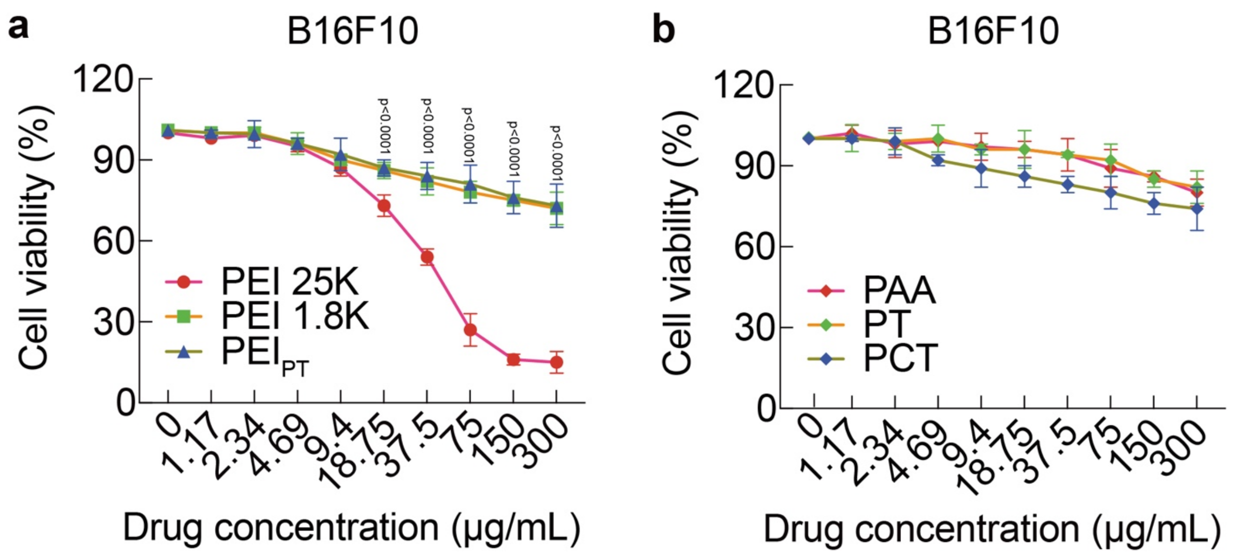

Supplementary Fig. 8. MTT assay of different materials. Data are presented as the mean ± s.d. (n = 3 biological replicates per group) and statistically analyzed using one-way ANOVA and Tukey's tests.

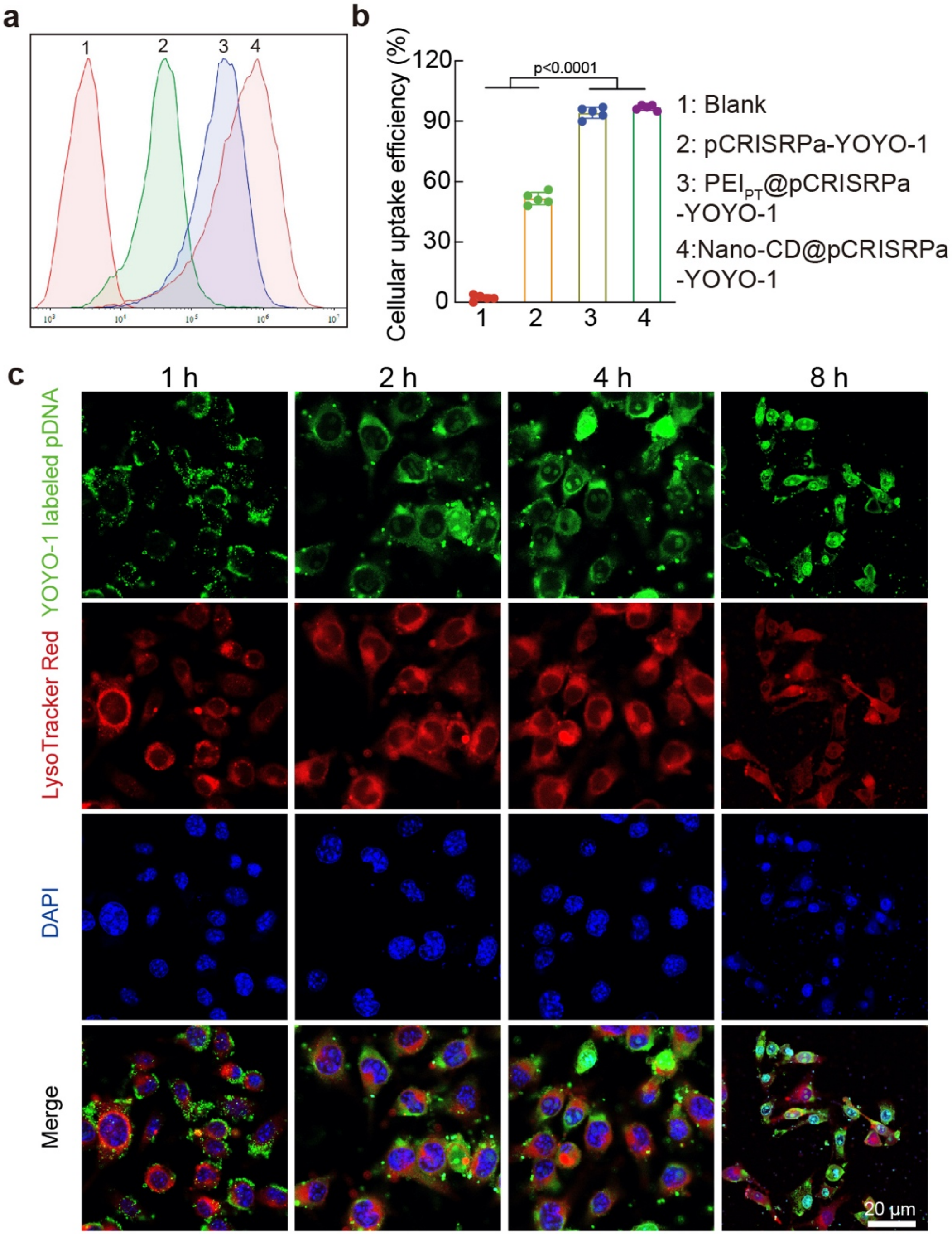

Supplementary Fig. 9. Cell uptake analysis. a, **b**, Representative cell uptake of different agents by FCM. Data are presented as the mean ± s.d. (n = 5 biological replicates per group) and statistically analyzed using one-way ANOVA and Tukey's tests. **c**, Intracellular tracking of Nano-CD in B16F10 cells. Endosomes and lysosomes were stained with LysoTracker Red (red), plasmids were labeled with YOYO-1 (green), and nuclei were stained with Hoechst 33342 (blue). (n = 3. Scale bar: 20 μm).

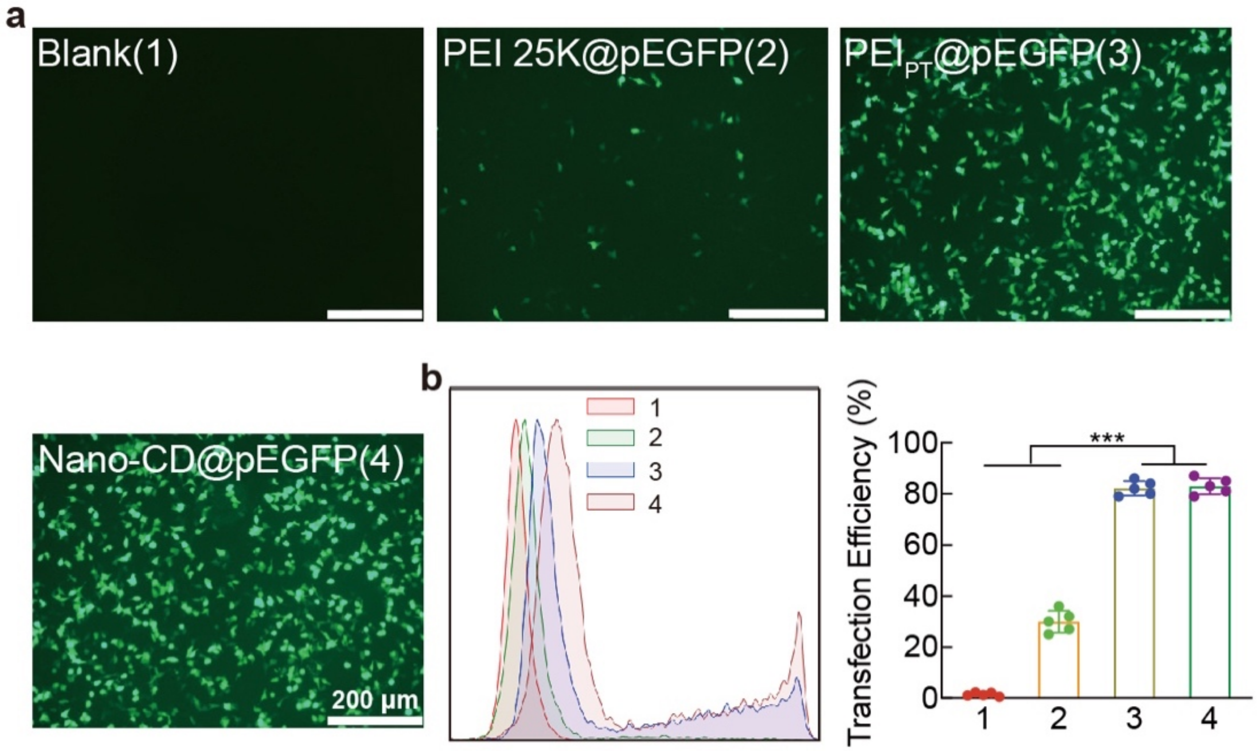

Supplementary Fig. 10. Transfection efficiency analysis. Representative images of GFP^+ B16F10 cells (green) by fluorescence microscope (Scale bar: 200 μm) and quantitative analysis of transfection efficiency by FCM. Data are presented as the mean ± s.d. (n = 5 biological replicates per group) and statistically analyzed using one-way ANOVA and Tukey's tests.

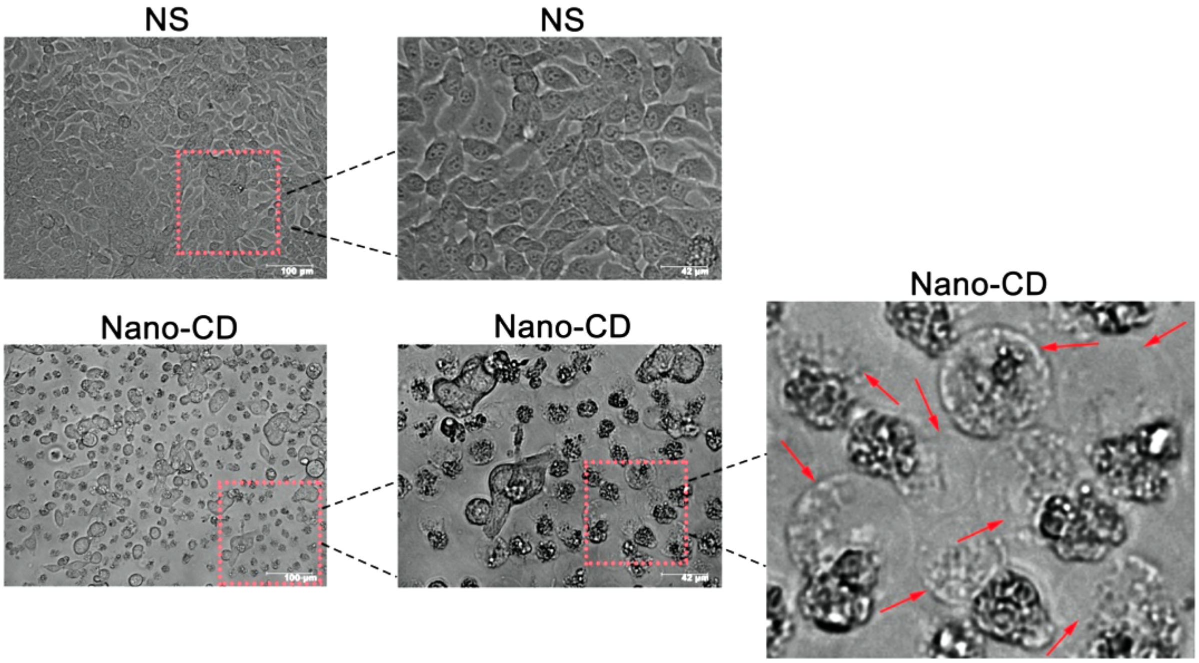

Supplementary Fig. 11 The morphological images of pyroptosis by CLSM. (n = 3. Scale bar: 42 μm).

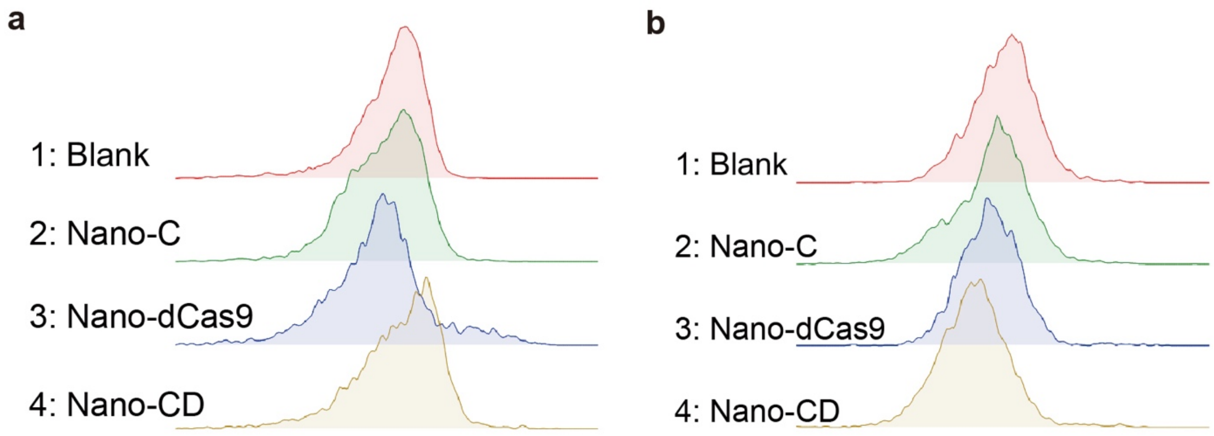

Supplementary Fig. 12. Detection of endogenous adjuvant effectors. a, CRT expression tested by FCM (n = 4). **b**, The HMGB1 expression analyzed by FCM (n = 4).

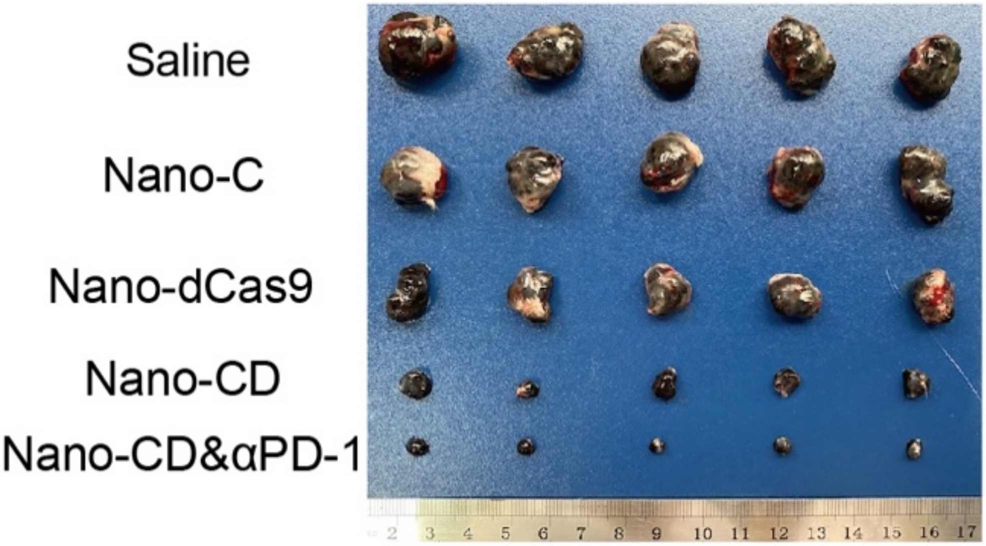

Supplementary Fig. 13. Representative pictures of B16F10 tumors after 24 days therapy.

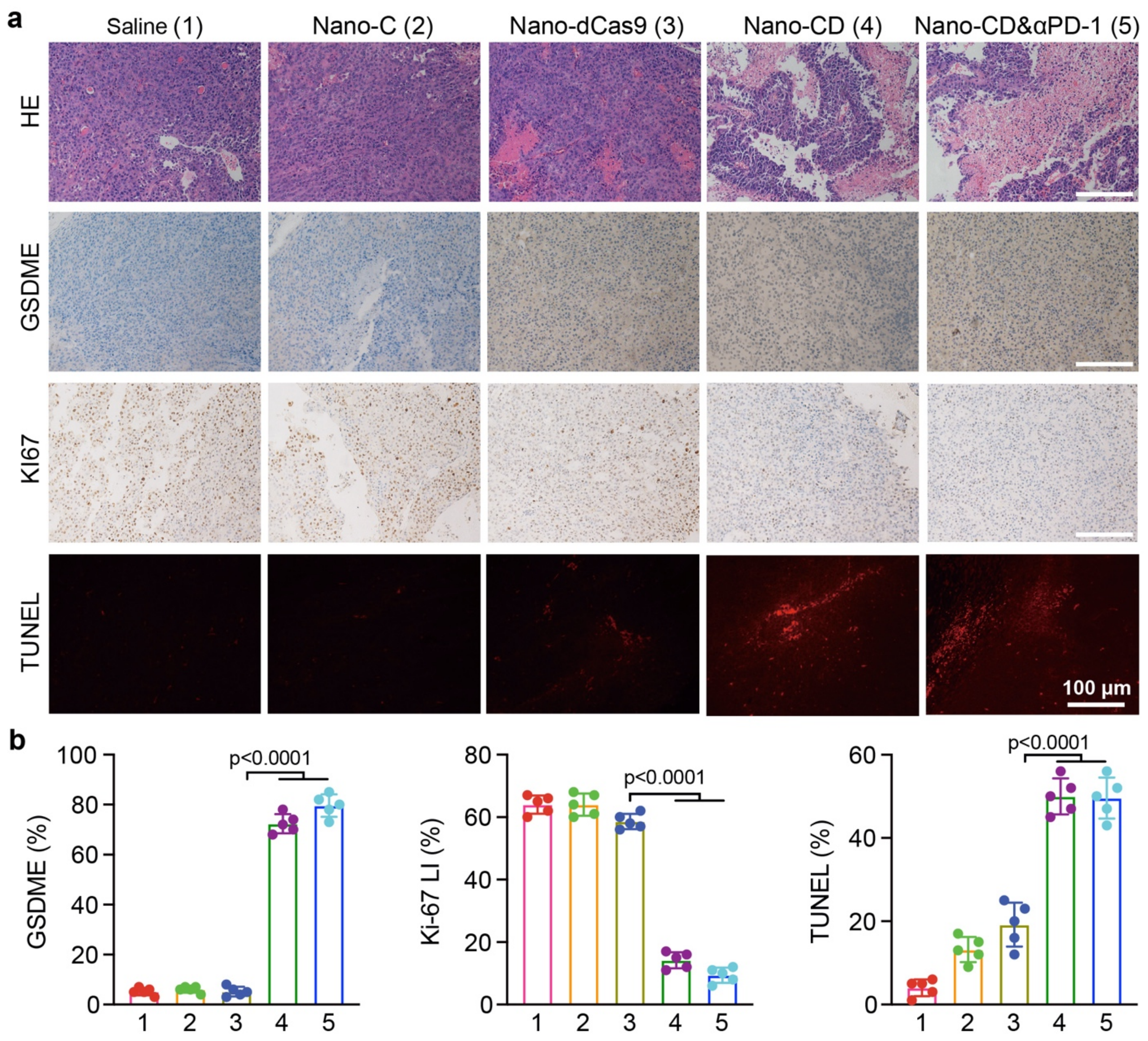

Supplementary Fig. 14. H&E staining and IHC analysis. a, Images of tumor sections stained with HE, GSDME (brown), Ki67 (brown) and TUNEL (red) by fluorescence microscope (Scale bar: 100 μm). **b**, Quantitative analysis of GSDME expression, Ki67 LI and apoptosis index of per group. Data are presented as the mean ± s.d. (n = 5 biological replicates per group) and statistically analyzed using one-way ANOVA and Tukey's tests.

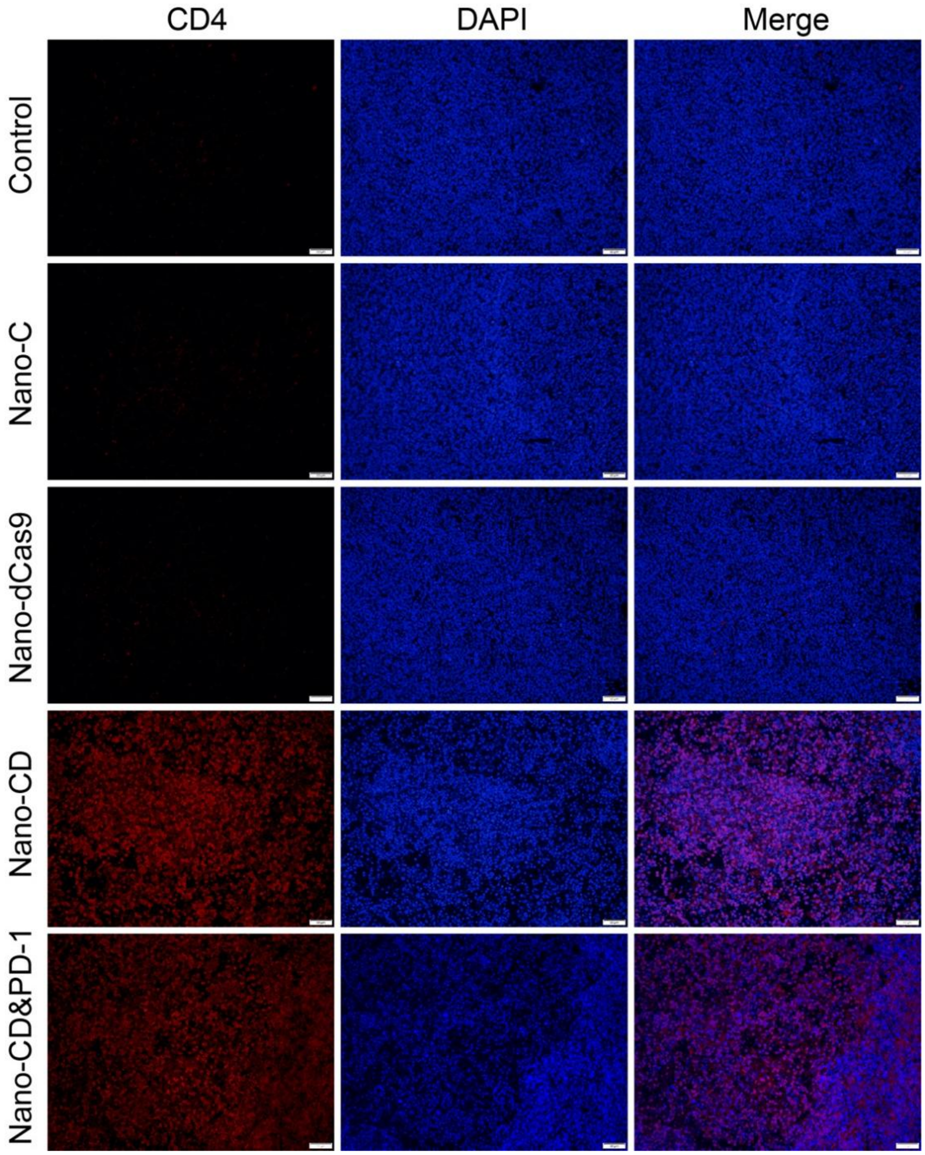

Supplementary Fig. 15. Immunofluorescence staining of $CD4^+$ T cells. $CD4^+$ T cells (red); nucleus (blue). (n = 3. Scale bar: 50 μm).

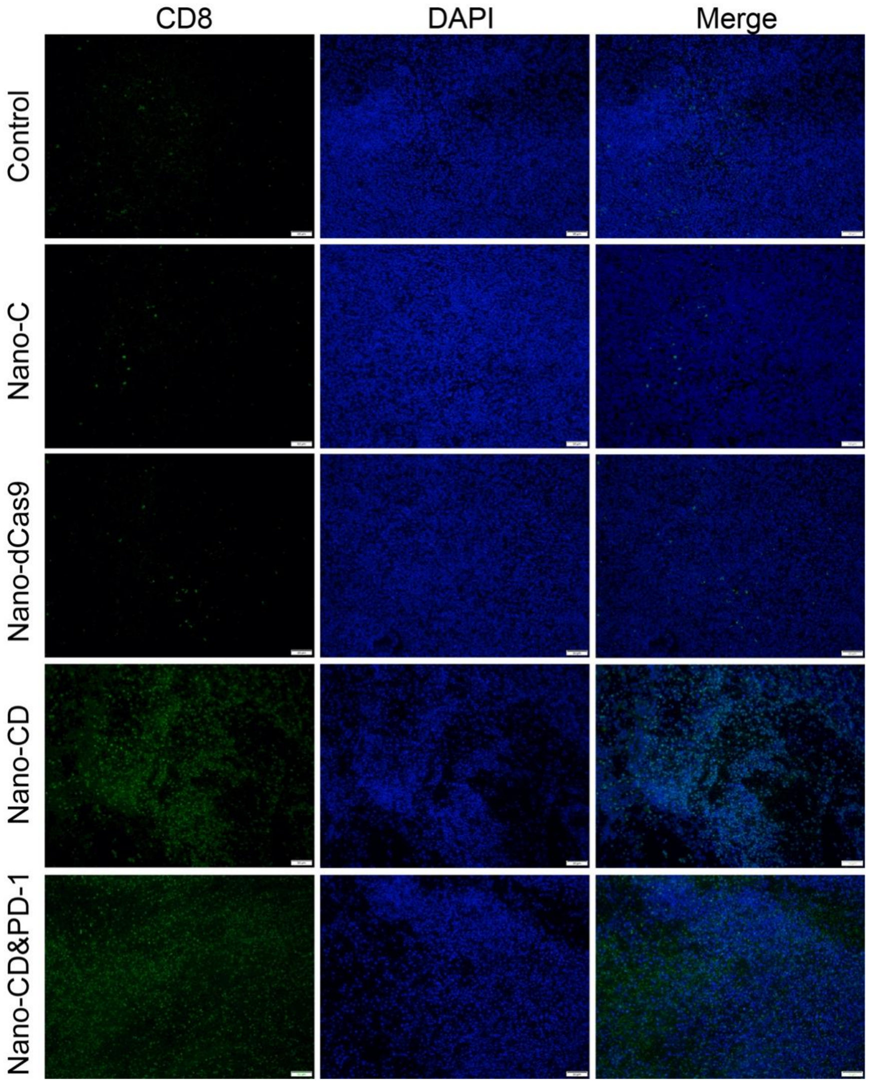

Supplementary Fig. 16. Immunofluorescence staining of $CD8^+$ T cells. $CD8^+$ T cells (green); nucleus (blue). (n = 3. Scale bar: 50 μm).

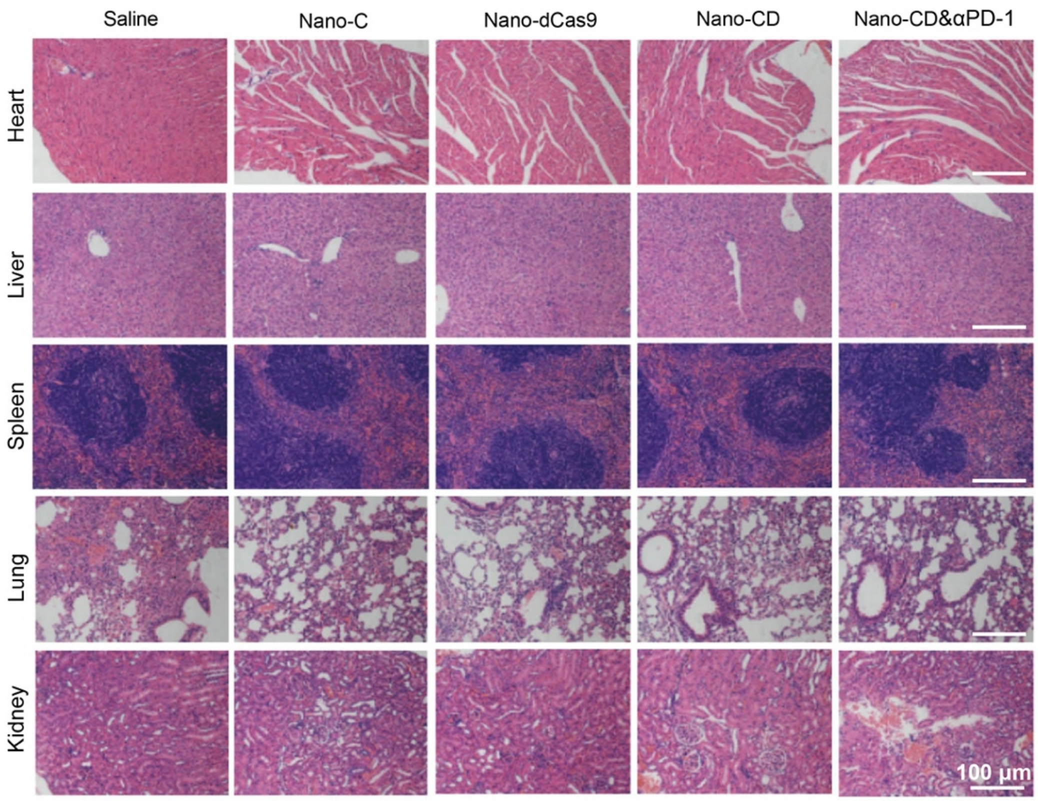

Supplementary Fig. 17. Evaluation of side-effect of Nano-CD in major organs of mice after different treatment. (n = 3. Scale bar: 100 μm).

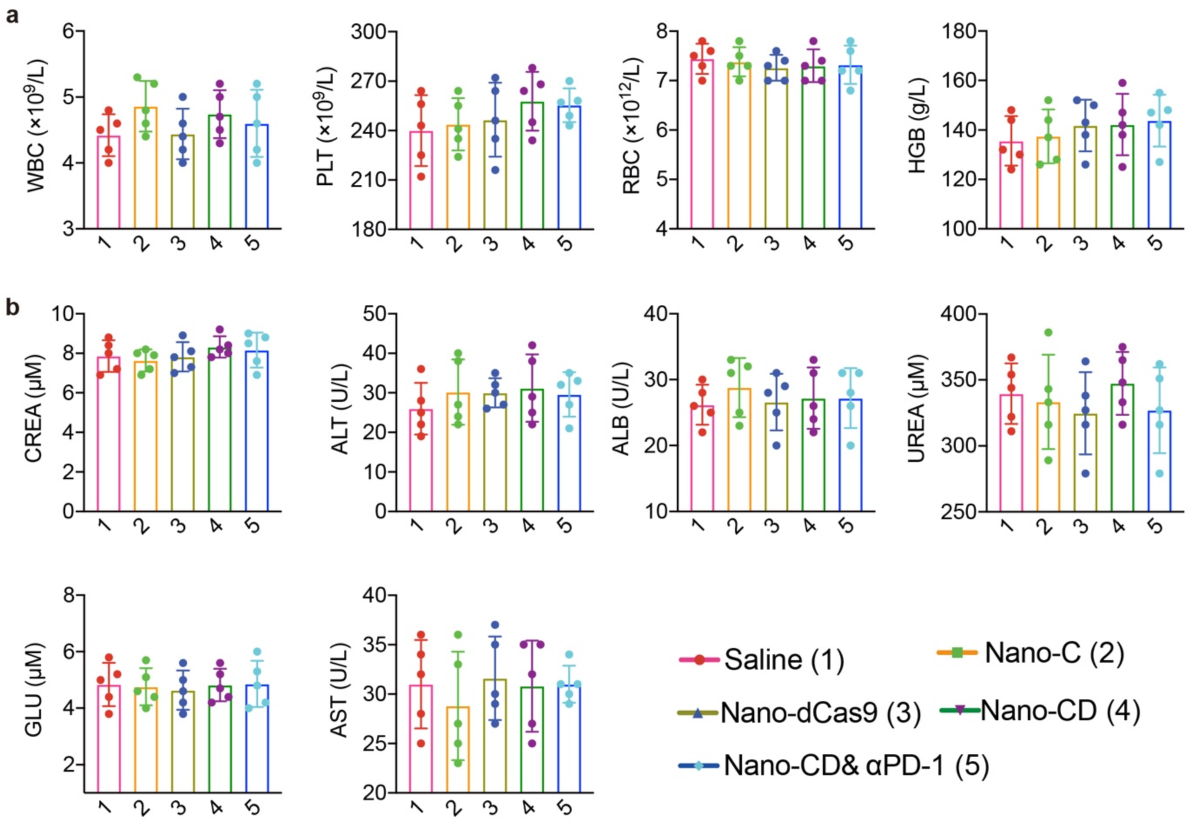

Supplementary Fig. 18. CBC test (a) and blood chemistry profile analysis (b) after treatment with different agents. Data are presented as the mean ± s.d. (n = 5 biological replicates per group)

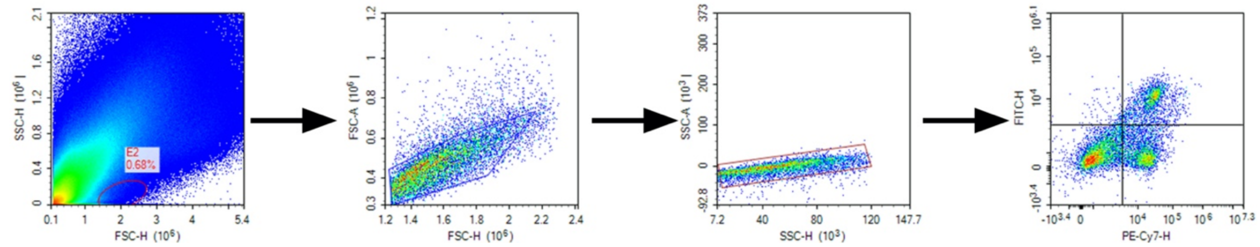

Supplementary Fig. 19. Representative scatter plots and gating information derived from analysis of $CD4^+$ T cells in tumor tissue.

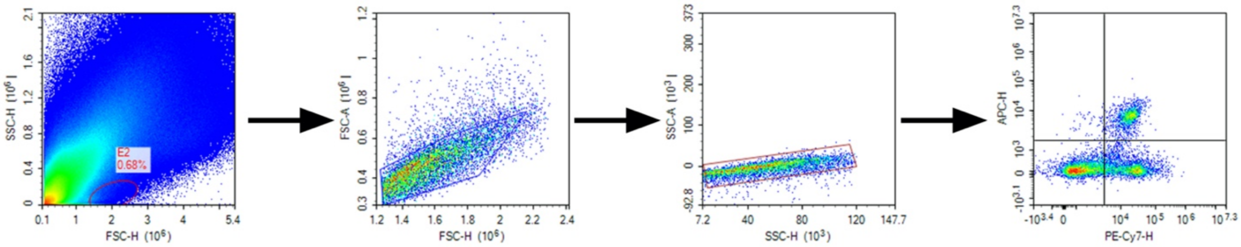

Supplementary Fig. 20. Representative scatter plots and gating information derived from analysis of CD8^{+} T cells in tumor tissue.

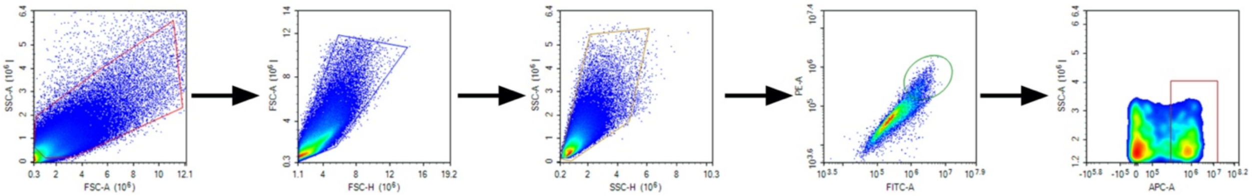

Supplementary Fig. 21. Representative scatter plots and gating information derived from analysis of CD206$^+$ macrophages in tumor tissue.

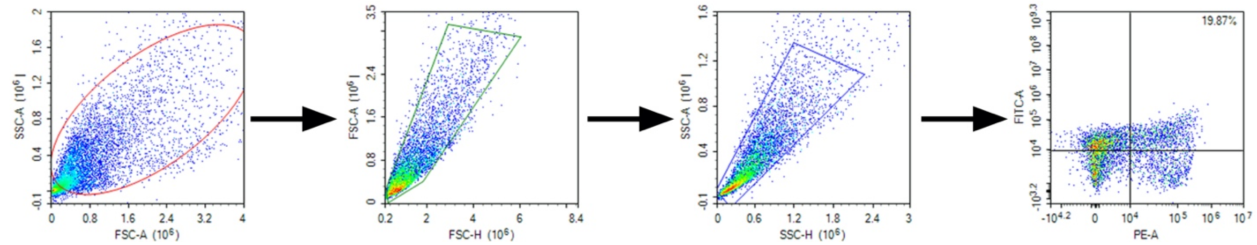

Supplementary Fig. 22. Representative scatter plots and gating information derived from analysis of T_{EM} cells in tumor tissue.

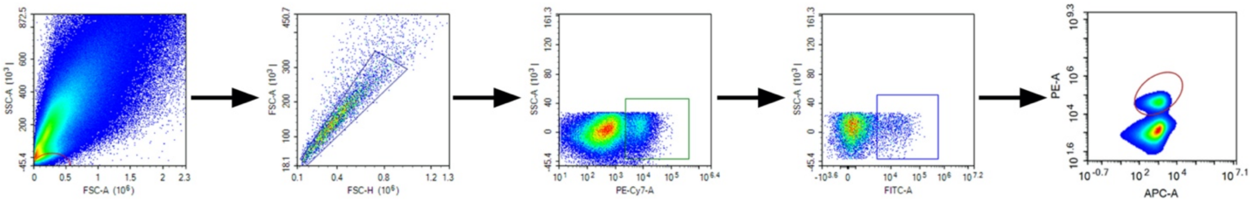

Supplementary Fig. 23. Representative scatter plots and gating information derived from analysis of T_{EM} cells in tumor tissue.

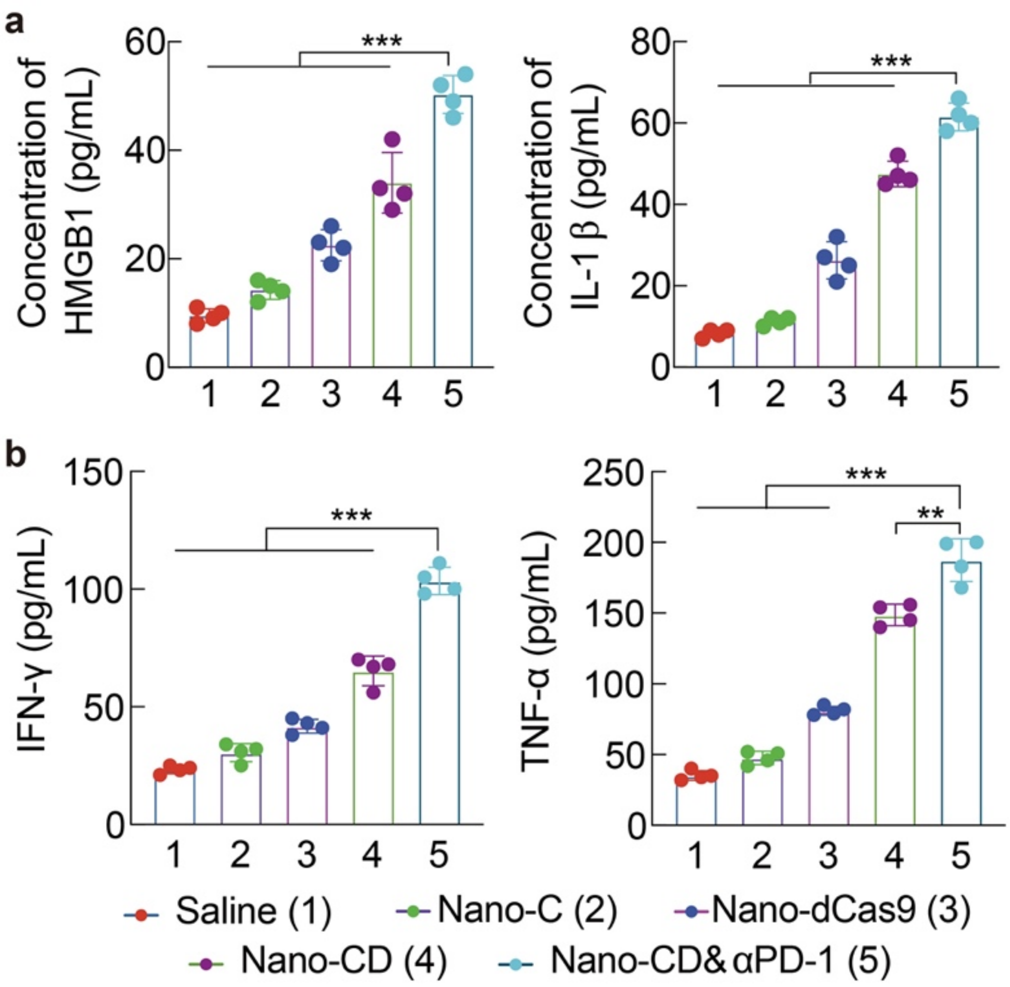

Supplementary Fig. 24. HMGB1 and cytokine concentrations in serum. a, The HMGB1 and IL-1β in serum by ELISA per group. **b**, Level of IFN-γ and TNF-α cytokines in serum per group. In panels **a** and **b**, data are presented as the mean ± s.d. (n = 4 biological replicates per group) and statistically analyzed using one-way ANOVA and Tukey's tests.

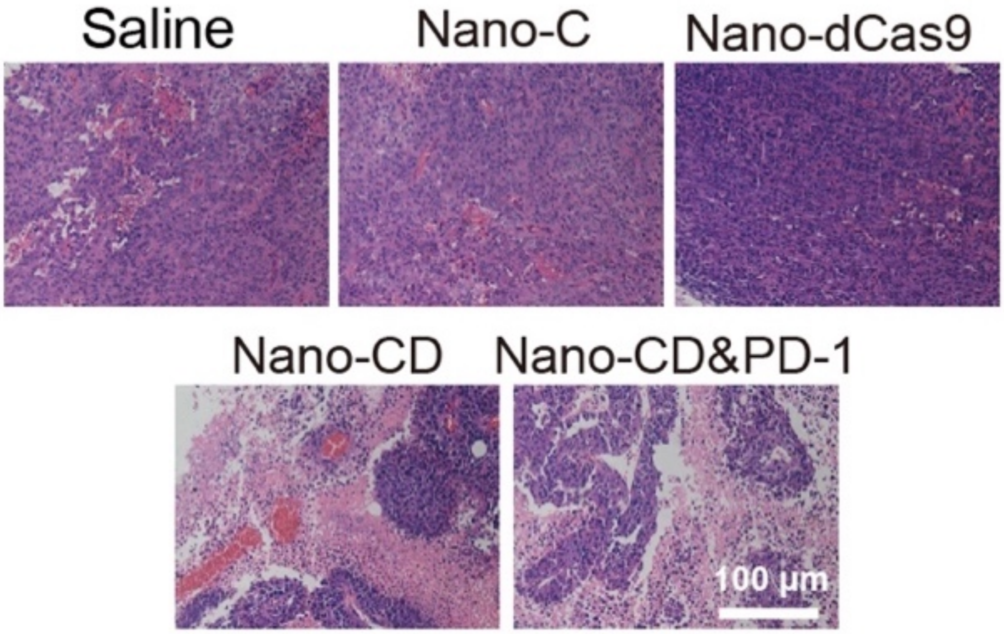

Supplementary Fig. 25. The H&E staining of tumor sections. (n = 3. Scale bar: 100 μm).

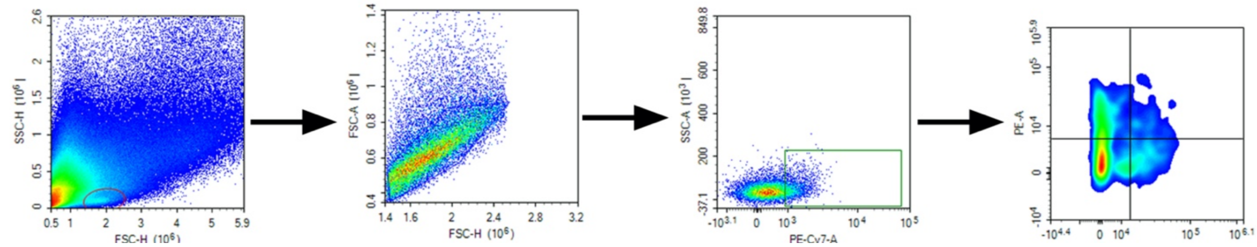

Supplementary Fig. 26. Representative scatter plots and gating information derived from analysis of CD69^{+} T cells in tumor tissue.

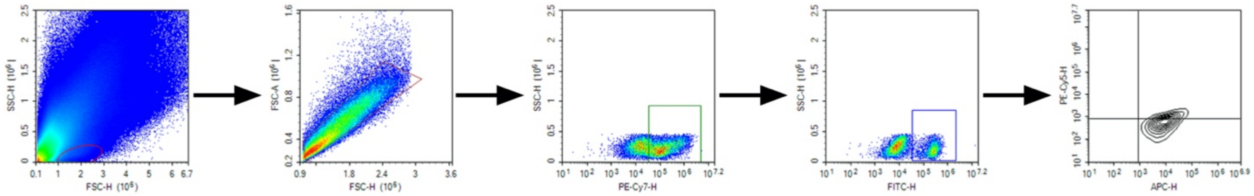

Supplementary Fig. 27. Representative scatter plots and gating information derived from analysis of Treg in tumor tissue.

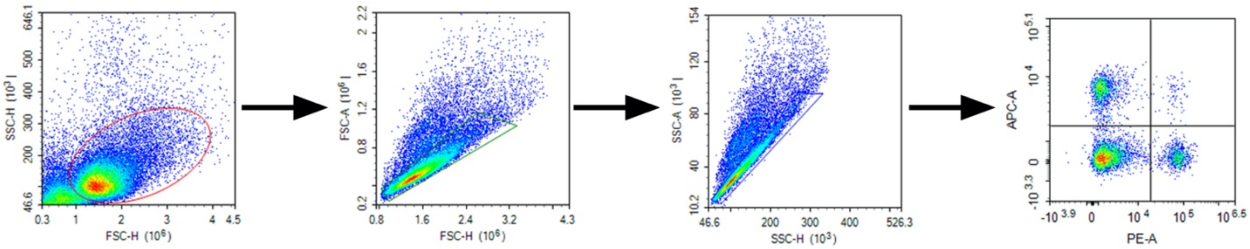

Supplementary Fig. 28. Representative scatter plots and gating information derived from analysis of CD4⁺ and CD8⁺ T cells in spleens.

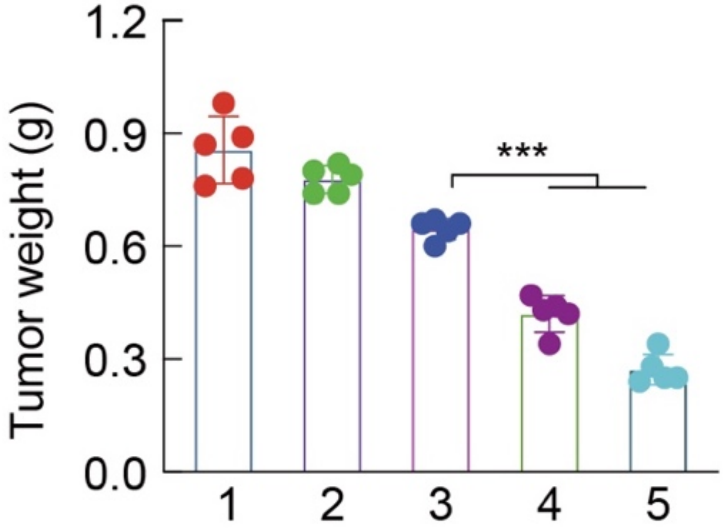

Supplementary Fig. 29. Profile of lung weight in each group. Data are presented as the mean ± s.d. (n = 5 biological replicates per group) and statistically analyzed using one-way ANOVA and Tukey's tests. (1: Saline, 2: Nano-C, 3: Nano-dCas9, 4: Nano-CD, 5: Nano-CD&αPD-1).

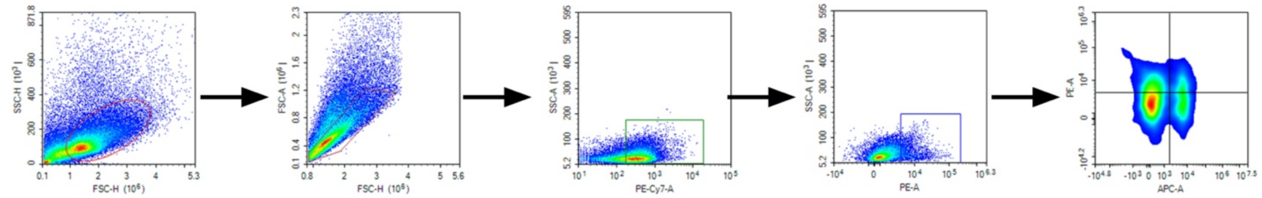

Supplementary Fig. 30. Representative scatter plots and gating information derived from analysis of CD69^{+} T cells in spleens.

Supplementary. Table 1. Modification efficiency of amino acid on $PEI_{PT}n$

**Polymer**	**Number of Phe + Tyr**	**X**	**Y**
**$PEI_{PT}2$**	1 + 1	0.2	1.25%
**$PEI_{PT}4$**	2 + 2	0.9	5.62%
**$PEI_{PT}8$**	4 + 4	2.3	14.34%
**$PEI_{PT}16$**	8 + 8	3.6	22.50%
**$PEI_{PT}20$**	10 + 10	5.5	34.38%
**$PEI_{PT}24$**	12 + 12	5.7	35.63%

X is the average number of Phe and Tyr on each PEI 1.8K molecular.

Y is the percentage of amino groups on prepared $PEI_{PT}n$.
