# Supplementary material for: A cooperative nano-CRISPR scaffold potentiates immunotherapy via activation of tumour-intrinsic pyroptosis
Source: Nat Commun. 2023 Feb 11;14:779. doi: 10.1038/s41467-023-36550-9 (PMC9922300; doi:10.1038/s41467-023-36550-9)
Supplement: Supplementary file 3 — Reporting Summary [file 41467_2023_36550_MOESM3_ESM.pdf]

nature portfolio

Corresponding author(s): Prof. Changyang Gong

Last updated by author(s): Jan 23, 2023

Reporting Summary

Nature Portfolio wishes to improve the reproducibility of the work that we publish. This form provides structure for consistency and transparency in reporting. For further information on Nature Portfolio policies, see our Editorial Policies and the Editorial Policy Checklist.

Statistics

For all statistical analyses, confirm that the following items are present in the figure legend, table legend, main text, or Methods section.

n/a	Confirmed	
☐	☒	The exact sample size (*n*) for each experimental group/condition, given as a discrete number and unit of measurement
☐	☒	A statement on whether measurements were taken from distinct samples or whether the same sample was measured repeatedly
☐	☒	The statistical test(s) used AND whether they are one- or two-sided<br>*Only common tests should be described solely by name; describe more complex techniques in the Methods section.*
☒	☐	A description of all covariates tested
☒	☐	A description of any assumptions or corrections, such as tests of normality and adjustment for multiple comparisons
☐	☒	A full description of the statistical parameters including central tendency (e.g. means) or other basic estimates (e.g. regression coefficient) AND variation (e.g. standard deviation) or associated estimates of uncertainty (e.g. confidence intervals)
☐	☒	For null hypothesis testing, the test statistic (e.g. *F*, *t*, *r*) with confidence intervals, effect sizes, degrees of freedom and *P* value noted<br>*Give P values as exact values whenever suitable.*
☒	☐	For Bayesian analysis, information on the choice of priors and Markov chain Monte Carlo settings
☒	☐	For hierarchical and complex designs, identification of the appropriate level for tests and full reporting of outcomes
☒	☐	Estimates of effect sizes (e.g. Cohen's *d*, Pearson's *r*), indicating how they were calculated

Our web collection on statistics for biologists contains articles on many of the points above.

Software and code

Policy information about availability of computer code

Data collection	Malvern Zetasizer Nano, UV-2600 spectrophotometer, FITR spectra Nicolet NEXUS 470, Zeiss confocal microscope, Hitachi SU-70 SEM microscope, IVIS Lumina LT Series III.
Data analysis	Flow cytometry results were analyzed by NovoExpress 1.5.6. Images were analyzed by Image J V1.8.0. NMR spectra were processed by Mestre Nova 14.<br>Zeta potential and size were analyzed using Zetasizer 6.34. In vivo fluorescence intensity was analyzed by living Image 4.5. Statistical calculations were performed using Graphpad Prism 8.

For manuscripts utilizing custom algorithms or software that are central to the research but not yet described in published literature, software must be made available to editors and reviewers. We strongly encourage code deposition in a community repository (e.g. GitHub). See the Nature Portfolio guidelines for submitting code & software for further information.

Data

Policy information about availability of data

All manuscripts must include a data availability statement. This statement should provide the following information, where applicable:

- Accession codes, unique identifiers, or web links for publicly available datasets
- A description of any restrictions on data availability
- For clinical datasets or third party data, please ensure that the statement adheres to our policy

The data that support the findings of this study are available within the article and its Supplementary Information files. Data generated in this study are provided in the Source Data file.

Human research participants

Policy information about studies involving human research participants and Sex and Gender in Research.

Reporting on sex and gender	N/A
Population characteristics	N/A
Recruitment	N/A
Ethics oversight	N/A

Note that full information on the approval of the study protocol must also be provided in the manuscript.

Field-specific reporting

Please select the one below that is the best fit for your research. If you are not sure, read the appropriate sections before making your selection.

☒ Life sciences ☐ Behavioural & social sciences ☐ Ecological, evolutionary & environmental sciences

For a reference copy of the document with all sections, see nature.com/documents/nr-reporting-summary-flat.pdf

Life sciences study design

All studies must disclose on these points even when the disclosure is negative.

Sample size	Sample size was chosen to ensure reproducibility of the experiments in accordance with the replacement, reduction and refinement principles of animal ethics regulation. Sample sizes employed in this study were referenced previously published studies (Nature Nanotechnology 2021, 16(1): 103-113).
Data exclusions	No data was excluded in this study.
Replication	Dara are presented as means and SD of at least 3 independent experiments. All experimental findings were reliably reproduced. All experiments were performed as technical or biological replications as appropriate for the experiment design. Details of experimental replicates are given in the figure legends.
Randomization	All samples were randomly allocated into experimental groups.
Blinding	No blinding was used throughout experiments. The investigators should keep careful track of protocols because that most of the experiments needed multiple treatments (including formulation, cells or mouse tumor treatment, sample collection, and so on). Hence, it would be difficult to blind the investigators to group allocation during data collection and analysis.

Reporting for specific materials, systems and methods

We require information from authors about some types of materials, experimental systems and methods used in many studies. Here, indicate whether each material, system or method listed is relevant to your study. If you are not sure if a list item applies to your research, read the appropriate section before selecting a response.

Materials & experimental systems

n/a	Involved in the study
☐	☒ Antibodies
☐	☒ Eukaryotic cell lines
☒	☐ Palaeontology and archaeology
☐	☒ Animals and other organisms
☒	☐ Clinical data
☒	☐ Dual use research of concern

Methods

n/a	Involved in the study
☒	☐ ChIP-seq
☐	☒ Flow cytometry
☒	☐ MRI-based neuroimaging

Antibodies

Antibodies used

Anti-Mouse CD40 (biolegend, #124612, 100 ×), Anti-Mouse CD80 (biolegend, #104706, 100 ×), Anti-Mouse CD86 (biolegend, #105113, 100 ×), and Anti-Mouse CD83 (biolegend, #121518, 100 ×), Rabbit Anti-HMGB1 antibody (abcam,#ab79823, 1000 ×), Anti-Calreticulin antibody (abcam, #ab196159, 1000 ×), Anti-GSDME antibody (abcam, #ab215191, 1000 ×), Anti-Ki67 antibody (affinity, #AF0198, 100 ×), Anti-Mouse CD3 (biolegend, #100220, 100 ×), Anti-Mouse CD4 (biolengd, #100510, 100 ×), Anti-Mouse CD8 (biolengd, #100714, 100 ×), Anti-Mouse F4/80 (biolengd, #123109, 100 ×), Anti-Mouse CD206 (biolengd, #141708, 100 ×), and Anti-Mouse CD11c (biolengd, #117307, 100 ×), Anti-Mouse CD25 (biolengd, #102808, 100 ×), Anti-Mouse Foxp3 (biolengd, #126408, 50 ×), Anti-Mouse CD69 (biolengd, #104508, 100 ×), Anti-Mouse CD44 (biolengd, #103012, 100 ×), Anti-Mouse CD62L (biolengd, #104412, 100 ×), Horseradish peroxidase (HRP)-labeled goat anti-mouse secondary antibody (beyotime, #A0216, 10000 ×), Horseradish peroxidase (HRP)-labeled goat anti-rabbit secondary antibody (beyotime, #A0208, 10000 ×)

Validation

1. Anti-Mouse CD40 (124612): https://www.biolegend.com/en-us/products/apc-anti-mouse-cd40-antibody-4984
2. Anti-Mouse CD80 (104706): https://www.biolegend.com/en-us/products/fitc-anti-mouse-cd80-antibody-41
3. Anti-Mouse CD86 (105113): https://www.biolegend.com/en-us/products/apc-anti-mouse-cd86-antibody-2342
4. Anti-Mouse CD83 (121518): https://www.biolegend.com/en-us/products/pe-cyanine7-anti-mouse-cd83-antibody-12359
5. Rabbit Anti-HMGB1 antibody (ab79823): https://www.abcam.cn/hmgb1-antibody-epr3507-ab79823.html
6. Anti-Calreticulin antibody (ab196159): https://www.abcam.cn/alexa-fluor-647-calreticulin-antibody-epr3924-er-marker-ab196159.html
7. Anti-GSDME antibody (ab215191): https://www.abcam.cn/dfna5gsdme-antibody-epr19859-n-terminal-ab215191.html
8. Anti-Ki67 antibody (AF0198): http://www.affbiotech.com/goods-897-AF0198-Ki67_Antibody.html
9. Anti-Mouse CD3 (100220): https://www.biolegend.com/en-us/products/pe-cyanine7-anti-mouse-cd3-antibody-6060
10. Anti-Mouse CD4 (100510): https://www.biolegend.com/en-us/products/fitc-anti-mouse-cd4-antibody-480
11. Anti-Mouse CD8a (100714): https://www.biolegend.com/en-us/products/apc-cyanine7-anti-mouse-cd8a-antibody-2269
12. Anti-Mouse CD11b (101206): https://www.biolegend.com/en-us/products/fitc-anti-mouse-human-cd11b-antibody-347
13. Anti-Mouse F4/80 (123109): https://www.biolegend.com/en-us/products/pe-anti-mouse-f4-80-antibody-4068
14. Anti-Mouse CD206 (141708): https://www.biolegend.com/en-us/products/apc-anti-mouse-cd206-mmr-antibody-7425
15. Anti-Mouse CD11c (117307): https://www.biolegend.com/en-us/products/pe-anti-mouse-cd11c-antibody-1816
16. Anti-Mouse CD25 (101903): https://www.biolegend.com/en-us/products/pe-anti-mouse-cd25-antibody-129
17. Anti-Mouse Foxp3 (126408): https://www.biolegend.com/en-us/products/alexa-fluor-647-anti-mouse-foxp3-antibody-4662
18. Anti-Mouse CD69 (104508): https://www.biolegend.com/en-us/products/pe-anti-mouse-cd69-antibody-265
19. Anti-Mouse CD44 (103012): https://www.biolegend.com/en-us/products/apc-anti-mouse-human-cd44-antibody-312
20. Anti-Mouse CD62L (104412): https://www.biolegend.com/en-us/products/apc-anti-mouse-cd62l-antibody-381
21. Horseradish peroxidase (HRP)-labeled goat anti-mouse secondary antibody (beyotime, #A0216, 10000 ×)
22. Horseradish peroxidase (HRP)-labeled goat anti-rabbit secondary antibody (beyotime, #A0208, 10000 ×)

Eukaryotic cell lines

Policy information about cell lines and Sex and Gender in Research

Cell line source(s)

Mouse melanoma cell lines (B16F10), Human melanoma cell lines (A375) and Human renal epithelial cell line (293T) were obtained from American type culture collection (ATCC).

Authentication

These cell lines were morphologically confirmed.

Mycoplasma contamination

No mycoplasma contamination was found.

Commonly misidentified lines
(See ICLAC register)

The used cell lines were not listed in commonly misidentified lines in ICLAC register.

Animals and other research organisms

Policy information about studies involving animals; ARRIVE guidelines recommended for reporting animal research, and Sex and Gender in Research

Laboratory animals

C57BL6 and Balb/c nude mice, female, 5-6-week-old, 18 ~ 20 g. Animals were housed under SPF conditions in groups of 4–5 mice per cage, and maintained at a temperature of ~25 °C in a humidity-controlled environment with a 12 h light/dark cycle, with free access to standard food and water.

Wild animals	No wild animals were used in this study.
Reporting on sex	The experiment was designed without considering the sex of the mice, and female mice were selected to ensure gender uniformity.
Field-collected samples	No field-collected samples were used in this study.
Ethics oversight	All animal procedures were carried out under the guidelines approved by the Institutional Animal Care and Use Committee (IACUC) of Sichuan University (Chengdu, China).

Note that full information on the approval of the study protocol must also be provided in the manuscript.

Flow Cytometry

Plots

Confirm that:

☒ The axis labels state the marker and fluorochrome used (e.g. CD4-FITC).

☒ The axis scales are clearly visible. Include numbers along axes only for bottom left plot of group (a 'group' is an analysis of identical markers).

☒ All plots are contour plots with outliers or pseudocolor plots.

☒ A numerical value for number of cells or percentage (with statistics) is provided.

Methodology

Sample preparation	the tumors were collected for the preparation of single cell suspension. After filtration through sterile screen (70 µm), the cell suspensions with cell density of 1×105 /100 µL was stained with anti-PercpCy5.5-CD3, anti-FTTC-CD4, anti-APC-CD8, anti-FITC-CD11b, anti-PE-F4/80, anti-APC-CD206, and anti-PE-CD11c for 30 min at 4 ℃ . The CD25 and Foxp3 antibodies were stained according to the operation manual. After washed with PBS for twice, the components of tumor immune microenvironment per groups were measured by flow cytometry.
Instrument	ACEA NovoCyte 2060R
Software	NovoExpress
Cell population abundance	No cell sorting was performed.
Gating strategy	The preliminary FSC/SSC gates were determined by the blank cell samples.

☒ Tick this box to confirm that a figure exemplifying the gating strategy is provided in the Supplementary Information.
